# A stem delphinidan from the Caribbean region of Venezuela

**DOI:** 10.1186/s13358-021-00217-z

**Published:** 2021-03-03

**Authors:** Aldo Benites-Palomino, Andres E. Reyes-Cespedes, Gabriel Aguirre-Fernández, Rodolfo Sánchez, Jorge D. Carrillo-Briceño, Marcelo R. Sánchez-Villagra

**Affiliations:** 1 Paläontologisches Institut Und Museum, Universität Zürich, Karl-Schmid-Strasse 4, 8006 Zürich, Switzerland; 2 Departamento de Ciencias Física Y Geociencias, Universidad del Norte, Barranquilla, Colombia; 3 Museo Paleontológico de Urumaco, Urumaco, Estado Falcón Venezuela

## Abstract

The dense Miocene record of cetaceans is known from localities along the coasts of all continents, mostly in the northern Atlantic or the eastern Pacific regions, but Antarctica. Fossils from the Caribbean region are few and include of a couple of findings from Panama and Venezuela. Here, we report a partly complete skull from the Caujarao Formation (middle Miocene), Falcon State, Caribbean region of Venezuela. Our phylogenetic analyses indicate that the Caujarao specimen is a ‘stem delphinidan’, a group that includes several taxa of early diverging odontocetes whose phylogenetic affinities remain a matter of debate. The fossil record has shown that this group of stem delphinidans was taxonomically diverse, but displayed a somewhat homogeneous cranial patterning, with most of the variations being found within the mandible or tympanoperiotic characters. As other stem delphinidans the Caujarao odontocete displays an enlarged temporal fossa and a fairly symmetrical cranium. Because the skull is missing several key diagnostic characters due to the preservation state of the specimen, a more precise taxonomic identification is not possible. Despite this, the finding of this specimen highlights the importance of the fossil record from the Neogene of Venezuela, and the importance of the area to understand cetacean evolution in the proto-Caribbean.

## Introduction

The Caribbean Sea is one of the most unusual marine regions of the world, characterized by a low productivity and a relative high salinity (Mertens et al. 2009). Marine species diversity of this region is mostly endemic and rather new due to the fairly recent closure of the Central American Seaway during the Miocene; and the subsequent rise of the Panama land isthmus during the Pliocene (Jaramillo 2018; Stange et al. 2018). The Neogene fossil record of marine vertebrates in the Caribbean is mostly represented by abundant chondrichthyan and bony fish remains (Aguilera 2010; Laurito and Valerio 2011; Carrillo-Briceño et al. 2015a, b, 2019; Aguilera et al. 2016). In contrast, cetaceans have a scarce and restricted record on the Caribbean region, which includes a few depositional sequences from the Miocene of both Colombia (Castilletes Fm.) and Venezuela (Canture Fm., Querales Fm. and Codore Fm.), surrounding the Gulf of Venezuela (Aguirre-Fernández et al. 2017a, b). An additional locality near the Piña town in eastern Panama has yielded the most complete specimens of the region known so far: the iniid *Isthminia panamensis* and the kogiid *Nanokogia isthmia* (Pyenson et al. 2015; Velez-Juarbe et al. 2015). Despite this, the scarce fossil record greatly contrasts with the extant diversity of cetaceans from the southern Caribbean. So far, at least 30 species have been recorded in the area, being mostly delphinids or balaenopterids, but also including physeteroids and ziphiids (van Bree 1975; Luksenburg 2014).

During the middle-to-late Miocene the odontocete diversity worldwide included a wide array of sperm whales (Lambert et al. 2017a; Benites-Palomino et al. 2020), beaked whales (Bianucci et al. 2016), stem delphinidans (Peredo et al. 2018; Kimura and Hasegawa 2019), and several longirostrine taxa belonging to Eurhinodelphinidae and Platanistoidea (Bianucci et al. 2020). Most of these groups have been found in multiple localities across the world, especially in the case of stem delphinidans. The latter, were classically identified as “Kentriodontidae”, a group that includes a wide array of early diverging odontocetes that display an approximately symmetric cranium around the bony nares, obvious contact between both nasals and their corresponding premaxillae, and single rooted teeth (Barnes 1978). Among ‘kentriodontids’, four ‘subfamilies’ have been proposed: Kampholophinae, Kentriodontinae, Lophocetinae and Pithanodelphinae (Barnes 1985). However, most of the recent phylogenetic analyses agree that ‘Kentriodontidae’ is a paraphyletic or polyphyletic group of early odontocetes stem to crown Delphinida (Lambert et al. 2017b; Peredo et al. 2018; Kimura and Hasegawa 2019). Because of this, new interpretations of what a ‘kentriodontid’ is, have been preliminary proposed based both on mandibular, and tympanoperiotic characters (Peredo et al. 2018; Kimura and Hasegawa 2019). The overall phylogenetic relationships of ‘Kentriodontidae’ and other early delphinidan species can only be properly addressed with new fossil findings and analyses. Here, we report the occurrence of a stem delphinidan based on a partial skull from the Caujarao Formation from the Caribbean region of Venezuela (Figs. 3, 4) and discuss its phylogenetic affinities, as well as its implications for the proto-Caribbean region.

## Materials and methods

**Fig. 1:**
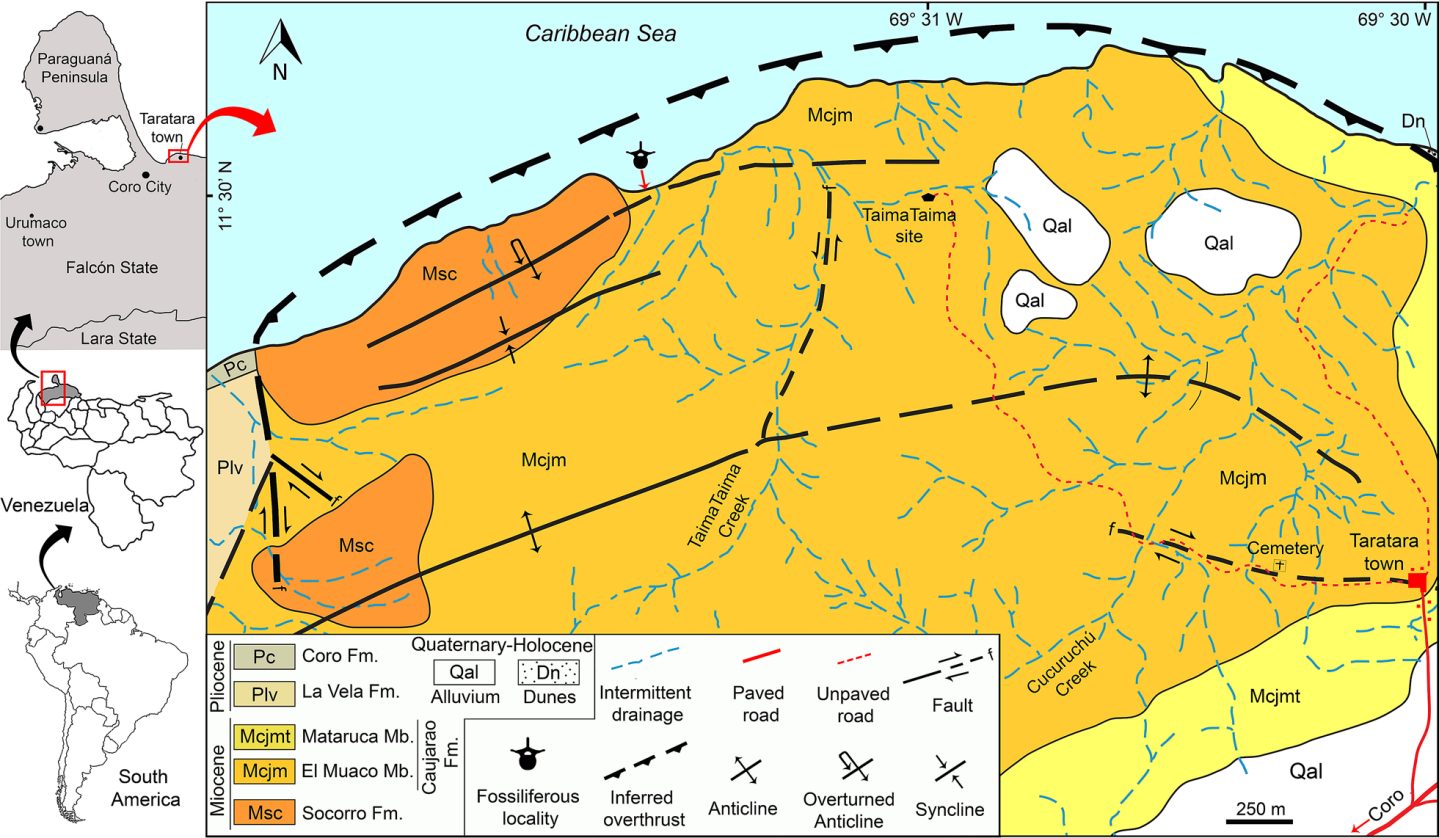
Map indicating the geographical location, general geology of the study area and fossiliferous locality of the specimen MTT-V-558

The cranial remains reported here were found on a coastal locality northeast of Coro city, Falcón State (Fig. 1; 11° 29′ 55′′ N, 69° 31′55′′ W). The specimen was collected in 2020 by two of the authors (R. Sanchez & A. Reyes) and is permanently housed at the paleontological collection of the Museo Ángel Segundo López, Taratara, Falcón State, Venezuela (MTT-V).

*Phylogenetic analysis*—In order to test the possible affinities of the Caujarao delphinidan, subsequent phylogenetic analyses were carried out (Fig. 5). The specimen was coded and included into an unmodified version of the matrix of Lambert et al. (2020). Following previous analysis, a molecular backbone constrain based on McGowen et al. (2019) was enforced, and nine fragmentary taxa were removed. A posterior parsimony analysis was performed based only on cranial characters, omitting tympanoperiotic and soft tissue characters (199–324) in order to test the support of the groups by solely using cranial characters. The third and fourth analyses were carried out following Viglino et al. (2020), performing a standard parsimony analysis, and an implied weighting, using the values of k = 20. In all cases the matrix was handled using Mesquite (Maddison and Maddison 2015) and the phylogenetic analyses were carried out in PAUP (Swofford 2003).

*Institutional abbreviations*—MTT-V, Museo Ángel Segundo López, Taratara, Falcón State, Venezuela; PIMUZ, Palaeontological Institute and Museum at the University of Zurich.

## Geological and geographical settings

The Caujarao Formation crops out in the Coro–La Vela region, reaching a thickness of 1200 m in its type locality (Fig. 1), near the Caujarao town, Coro river (Vallenilla 1961); it overlays the middle Miocene Socorro Formation. In the type locality, the Caujarao Fm. is mainly characterized by mudstones and clays, with a lesser proportion of limestones and sandstones (Vallenilla 1961; González de Juana et al. 1980). A fully marine environment and a late Miocene age have been suggested for the Caujarao Formation based on mollusks, foraminifera and nanoplankton (González de Juana et al. 1980; Wozniak and Wozniak 1987). Three members have been recognized for the Caujarao Fm.: El Muaco (lower), Mataruca (middle) and Taratara (upper). Mollusks and foraminifera indicate that the El Muaco Member was deposited in a marine shallow environment with pelagic influence (Cavanahg de Petzall 1959; Vallenilla 1961; Wozniak and Wozniak 1987) during the Tortonian, as suggested by planktonic foraminifera (Carrillo et al. 2018, Fig. 29).

**Fig. 2:**
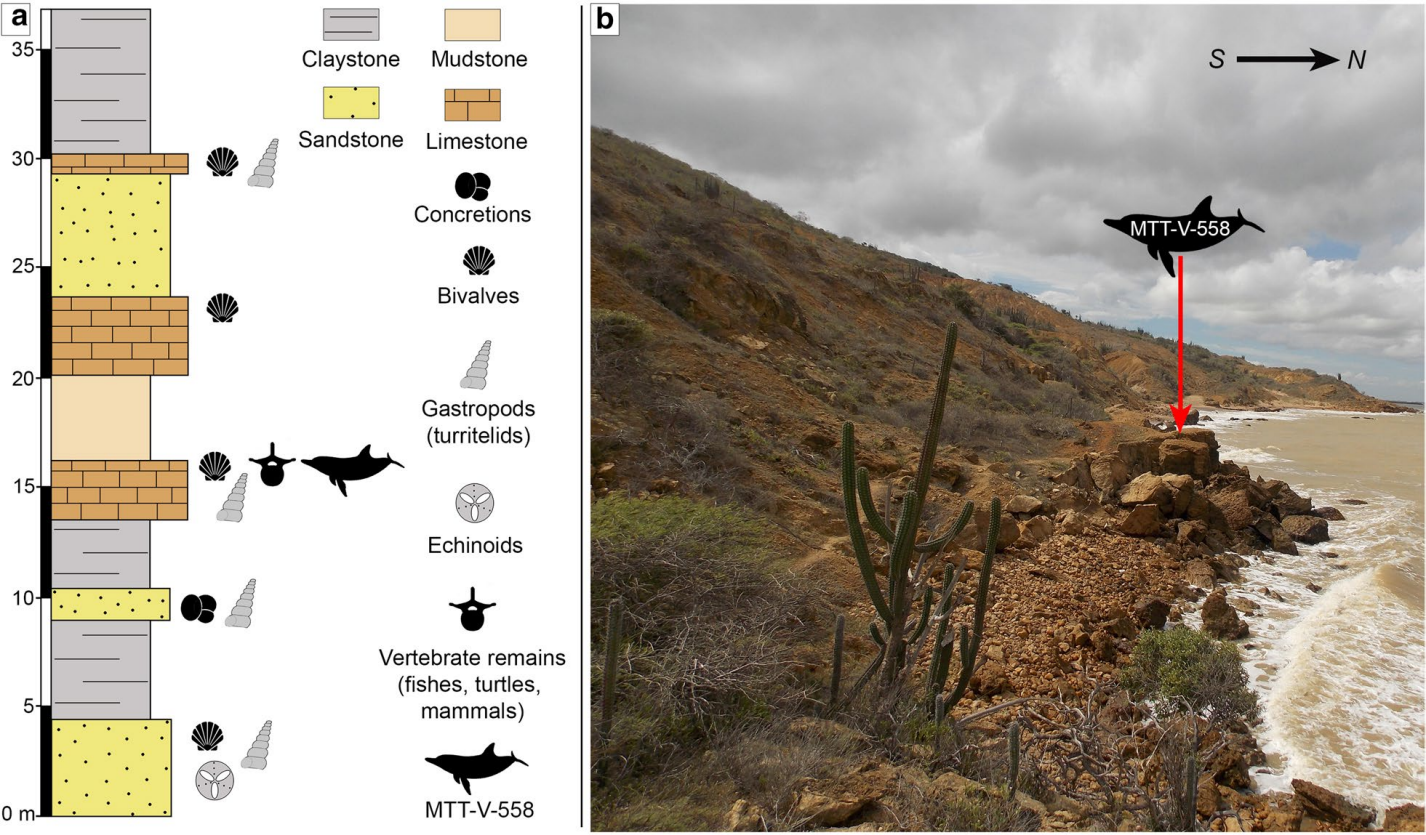
General stratigraphic section of the fossiliferous locality where the specimen MTT-V-558 was collected. The dolphin silhouette shows the fallen block where the specimen was found

The specimen MTT-V-558 was collected from a fallen block (Fig. 2b), which is inferred here as coming from the base of the El Muaco member, on the beach that surrounds the northern flank of La Vela anticline, northwest of Taratara town (Fig. 1). In the area, El Muaco Member overlaps the Socorro Formation. The outcrops of the latter represent a ‘geologic window’ on the crest of the main fold, as well as on the crest of an overturned fold located north of the main fold (Gonzales de Juana 1937; Cavanahg de Petzall 1959). The El Muaco member reaches a thickness of 690 m in its type locality (Quebrada El Muaco), with a lithology characterized mainly by mudstones and claystones with some limestones and sandstones well developed at the basal section of the member (Cavanahg de Petzall 1959; Vallenilla 1961).

A new stratigraphic section for the fossiliferous locality of the specimen MTT-V-558 is presented here (Fig. 2a, b). The section is approximately 37 m thick and it was raised from the strata that emerge at sea level, which have a dip of 46° south. Lithological similarities of the limestone layer referred at the 15 m of the stratigraphic section (Fig. 2a), and the fallen block where MTT-V-558 was found, suggest that the later comes from this limestone layer. Both, the layer (at the section) and the fallen block are characterized by a fossiliferous ochre limestone with abundant turritelids (*Turritella*) and pectinids. In the fallen block, other vertebrate remains that include ray teeth, and fragments of bony fishes, turtles, and indeterminate mammals were also found. From the base to the top, the first 14 m of the section are characterized by an alternation of claystones and sandstones, with the presence of turritelids and pectinids. The fossiliferous limestone that represents the base of the Muaco member (characterized by the abundance of *Turritella altilira*) lays on the top of the Socorro Formation (Cavanahg de Petzall 1959). Strata with a different lithology and characterized by the absence of abundant macrofossils, crop out just farther west from our new stratigraphic section. The lithology of the Socorro Formation in the La Vela anticline consists of gray shales interbedded with fossiliferous marls, sandstones and limestones. Future stratigraphic works could shed light on the stratigraphy of El Muaco Member in this the area of La Vela anticline, since in the area the only well-studied sections so far correspond to these of the type locality.

SYSTEMATIC PALEONTOLOGY

CETACEA Brisson, 1762

PELAGICETI Uhen, 2008

NEOCETI Fordyce and de Muizon, 2001

ODONTOCETI Flower, 1867

DELPHINIDA Muizon, 1988a, b

**Fig. 3:**
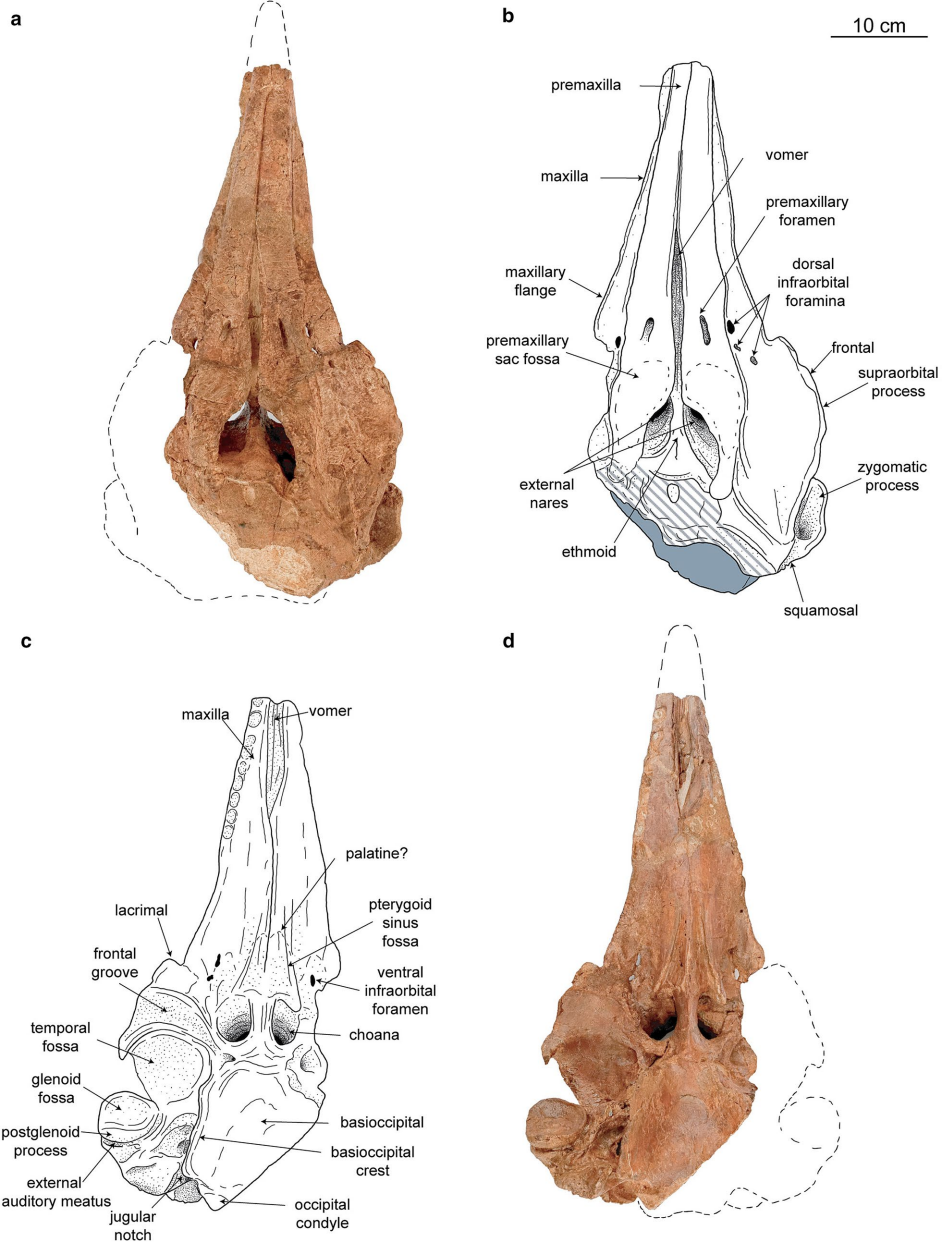
Caujarao delphinidan, MTT-V-558 referred skull in dorsal (**a**, **b**) and ventral (**c**, **d**) views. Dashed fill represents eroded bone, and gray areas indicate sediment

**Fig. 4:**
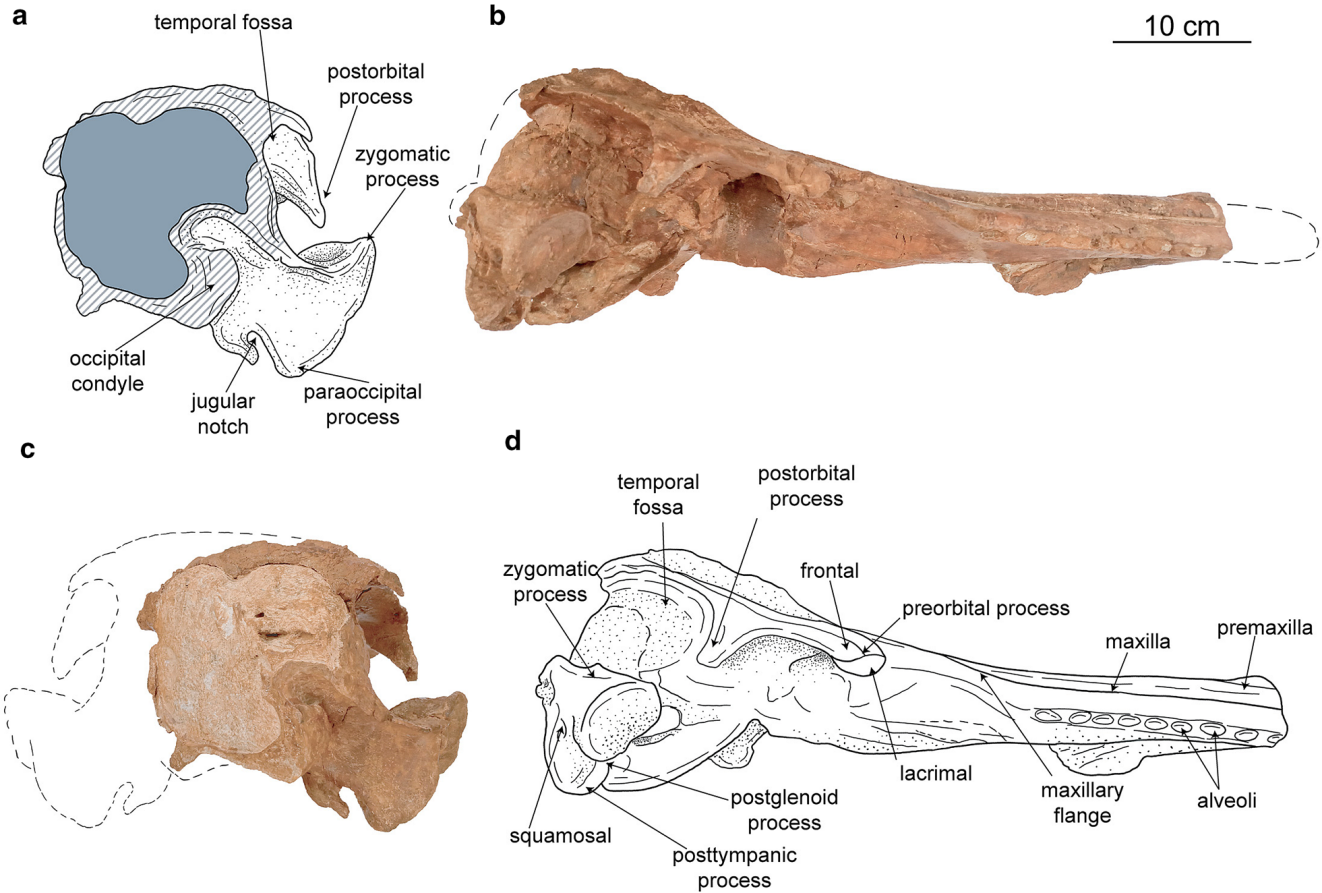
Caujarao delphinidan, MTT-V-558 referred skull in posterior (**a**, **c**) and lateral (**b**, **d**) views. Dashed fill represents eroded bone, and gray areas indicate sediment

Delphinida indet.

(Table 1, Figs. 3, 4).

**Table 1:** Measurements (in mm) of the referred skull of the Caujarao delphinidan MTT-V-558 (modified from Perrin 1975)

Dimensions	MTT-V-558
Condylobasal length	530+
Length of the rostrum	275+
Width of rostrum at base	160
Width of the rostrum at 60 mm anterior to the line across hindmost limits of antorbital notches	112
Width of rostrum at midlength	95
Width of premaxillae at midlength of the rostrum	70
Width of rostrum at 3⁄4 length, measured from posterior end	81
Height of rostrum at base	82
Height of rostrum at midlength	49
Height of rostrum at end	46
Distance from tip of rostrum to external nares	347+
Maximum width of the external right bony nare	27
Maximum width of the external left bony nare	31
Greatest preorbital width (width across preorbital processes)	240e
Greatest postorbital width (width across postorbital processes)	290e
Least supraorbital width	270e
Maximum width of external nares	71
Greatest width across zygomatic processes of squamosal	270e
Greatest width of premaxillae	127

*e* estimate, + measurement on incomplete element

*Referred specimen*—MTT-V-558 a partly preserved skull, lacking the posterior left part of the cranium and most of the vertex.

*Horizon and age*—MTT-V-558 comes from a fallen block, interpreted as belonging to the lowermost part of the Caujarao Fm. (late–middle Miocene) in the northern flank of La vela anticline (Fig. 1; 11° 29′ 53.9′′ N, 69° 31′57′′ W).

## Description

*General features of the skull*—The skull is partly eroded and several features of the tip of the rostrum, vertex, basicranium and left occipital region are missing. The referred skull has a preserved condylobasal length of 53 cm and an estimated bizygomatic width of 27 cm. Based on the equation by Pyenson and Sponberg (2011) an estimated body length of 2.63 m was calculated, being in the size range of extant *Tursiops truncatus*. The sutures between the well-ossified cranial bones are closed and, in some cases, ankylosed (e.g., occipital sutures), thus indicating that this skull corresponds to an adult individual. In lateral view, the rostrum is slightly transversely robust, with the premaxillae protruding dorsally, only in the anterior region. The external bony nares display a fairly asymmetrical outline, being antero-posteriorly longer than wide. It is not clear if this condition is a result of taphonomic processes, as the dorsalmost part of the vertex is missing, including both nasals. The temporal fossa is much well-developed than in more derived delphinidans, being deeper and extending to the occipital region.

*Premaxilla*—Along the dorsal surface of the rostrum the premaxillae are slightly convex. On the preserved anterior half of the rostrum, the premaxilla is notoriously wider than the maxilla in dorsal view, restricting the latter to a narrow strip laterally. At midlength of the preserved rostral region, the premaxillae are slightly deflected leftwards due to the taphonomic compression; however, these retain most of their width all across most of the anterior half of the rostrum. On the posterior half of the rostrum, the mesorostral groove opens as a narrow canal, anterior to the premaxillary foramen (Fig. 3a, b). Posterior to the premaxillary foramen, the mesorostral groove narrows for 4.8 cm before the anterior wall of the external bony nares. The premaxillary foramen has an antero-posteriorly elongated outline and it is located slightly anterior to the antorbital notch, which is followed by a posterior widening of the maxilla. On its posterior region, the premaxilla narrows laterally along the external bony nares, displaying a triangular shape in this region. On its posterior end the premaxillae projects slightly over the frontal, with a blunt ending. Anterolaterally to the external bony nares, the premaxilla forms a shallow depression with a triangular outline, interpreted as the premaxillary sac fossa (Fig. 3a, b). Ventrally the premaxilla is not exposed.

*Maxilla*—Dorsally, the maxilla is transversely convex along the rostrum, flattening posteriorly over the facial region. The dorsal exposure of the maxilla on the rostrum is mostly limited to its anterior third, where it is restricted to a narrow stripe. From a short distance (8.5 cm) anterior to the antorbital notch, the maxilla widens postero-laterally forming the lateral maxillary flange and reaching the same width as the premaxillae at the level of the antorbital notch. The antorbital notch displays a sharp ‘V’-shape outline. On the right side, in the area of the right antorbital notch, there are three small dorsal infraorbital foramina (Fig. 3a, b). The anterior end of the smallest foramen (0.3 cm in length) is located at the level of the antorbital notch. The largest foramen is located near the suture with the premaxilla, 0.8 cm anterior to the first foramen, and it has a length of 0.6 cm. The last and posteriormost foramen has a length of 0.4 cm and is located 0.8 cm posterior to the level of the antorbital notch. In dorsal view, the antorbital process displays a slight triangular profile. Along the facial region, the dorsal surface of the maxilla remains mostly flat, except for a small region near the antorbital process where it thickens over the frontal and the lacrimal. Posteriorly, the maximum width of the maxilla is reached at the level of the anterior wall of the external bony nares. In its posteriormost region, the ascending process of the maxilla narrows until the level of the posterior wall of the temporal fossa, exposing dorsally the floor of the temporal fossa, made by the squamosal (Fig. 3a, b). Ventrally, the maxilla is mostly flat to slightly convex along the rostrum. On the anterior third of the rostrum, the left maxilla is moderately displaced laterally due to compression, exposing ventrally the vomer. The alveoli are moderately sized (1.2 cm of average diameter), being transversely longer than wider (Fig. 3c, d). Only the preserved anterior half of the rostrum has functional alveoli, with the first posterior alveolus being located slightly anterior to the widening of the maxillary flange. The suture between the palatine and the maxilla is not visible due to fusion of both bones. The only hint of its position is the palatine sulcus which runs parallel to the sagittal plane along most of the palatal surface, slightly diverging posteriorly.

*Vomer/Ethmoid*—The vomer is ventrally exposed on the anterior third of the rostrum between the maxillae (Fig. 3c, d). On the dorsal region, part of the vomer is visible in dorsal view, due to the posteriorly open mesorostral groove. The nasal plate or septum is visible both in dorsal and ventral view. The external bony nares are located slightly more posteriorly than the choanae, which gives the nasal passages a moderate anteroventral orientation. Posteriorly, the suture between the vomer and the ethmoid is not clear due to fusion; despite this, most of the cribriform plate of the ethmoid should have formed the posterior wall of the nasal passages.

*Lacrimal*—The lacrimal is mostly restricted to the ventral and lateral regions of the skull, where it displays a sub-quadrangular outline (Fig. 4b, d). There are no hints of a preserved jugal. The preorbital process of the lacrimal is moderately robust and slightly ventrally projected. In ventral view, the surface of the lacrimal is mostly flat, lacking the development of a well-defined lacrimomaxillary fossa. The lacrimal only contributes to the most anteroventral portion of the ventral orbital crest, where the suture with the frontal lays along. In lateral view, the lacrimal is slightly thickened at midlength.

*Frontal*—Dorsally the exposure of the frontal is limited to a narrow strip of the supraorbital process lateral to the maxilla; and the external surface of the bone (*facies externa*; Mead and Fordyce 2009), exposed between the posteriormost region of the maxilla and the nuchal crest. Dorsally, the frontal is slightly covered by the premaxilla, posterolateral to the external bony nares. In lateral view, the dorsal surface of the surpraorbital process rises posterodorsally with an angle of about 30 degrees with main axis of the skull (Fig. 4b, d). The frontal retains its thickness all along its length, but it is slightly dorso-ventrally compressed posteriorly, when forming the roof of the temporal fossa. The frontal projects between the maxilla and the lacrimojugal, extending to approximately to midlength of the preorbital process. The postorbital process is antero-posteriorly narrow, displaying a boxy outline in its posteroventral end. The preorbital and postorbital processes reach ventrally the same level, somewhat higher than the dorsal limit of the rostrum. In ventral view, the frontal groove is ‘V’-shaped and forms an angle of 55 degrees with the main axis of the skull in its proximal region. Distally, it deviates laterally, drawing a curve in ventral view.

*Temporal/parietal*—The temporal fossa is antero-posteriorly longer than dorso-ventrally high and displays a trapezoidal outline in lateral view. The fossa is moderately deep, and its medial wall is mostly concave, flattening slightly posteriorly (Fig. 4b, d). Dorsally, the dorsal roof of the temporal fossa has a plate-like aspect, tapering posteriorly. The posterior limit of the temporal fossa is not preserved, not allowing to determine if the temporal crest extended posteriorly beyond the medial part of the occipital shield as in most stem delphinidans. However, the way the supramastoid crest raises posteriorly suggests that the temporal fossa was not significantly longer posteriorly.

*Squamosal*—The zygomatic process is anterodorsally short, not anteriorly elongated (Fig. 4b, d). Transversely, it displays a robust cross section, condition enhanced by the blunt anterior edge of it. Because the anteriormost tip of the zygomatic process is missing it is not clear whether if this was short or displayed a more elongated profile. The dorsomedial surface of the zygomatic process is mostly concave, with a moderately developed supramastoid crest lateral to it. Relative to the rest of the skull, the anteriormost end of the zygomatic process is located posterior to both the postorbital process of the frontal and the external bony nares; and below the ventral end of both the preorbital and the postorbital process of the frontal. In lateral view, the zygomatic process displays a robust triangular outline. Ventrally, the glenoid fossa is square-shaped, being rather shallow and slightly laterally oriented (Fig. 3c, d). The anterior wall of the glenoid fossa is missing, with the rest of the zygomatic process, thus preventing from discerning its full shape. The postglenoid process is robust and blunt ended. Posterior to it, the external auditory meatus is located between two well-ossified thick bony walls opening postero-laterally. The posttympanic process extends ventrally well below the level of the postglenoid fossa, displaying a square-shaped outline in lateral view (Fig. 4b, d). Ventrally, near the area of the basioccipital crest, only the base of the falciform process is preserved as a tiny rod (Fig. 3c, d). Posterior to it, the periotic fossa is somewhat deep, trapezoidal-shaped and anteromedially longer than wider.

*Occipital*—The occipital condyle protrudes moderately posteriorly, lacking a peduncle, and possessing a triangular outline in ventral view due to erosion. There is no hint of a preserved dorsal condyloid fossa as there is not bone preserved over the right occipital condyle (Fig. 4a, c). The paroccipital process extends well below the level of the condyle reaching the same level as the basioccipital crest. Ventrally, the basioccipital sutures are not well defined, due to the advanced degree of fusion with adjacent bones (Fig. 3c, d). The medial surface of the basioccipital in the ventral area is mostly flat to slightly concave. Only the right pharyngeal crest is preserved in its most dorsal portion, with small bone fragments that should correspond to the medial lamina of the pterygoid. Between the basioccipital crest and the paroccipital process the jugular notch opens as a moderately wide or deep incision, opening ventrolaterally (Fig. 4a, c).

## Results

**Fig. 5:**
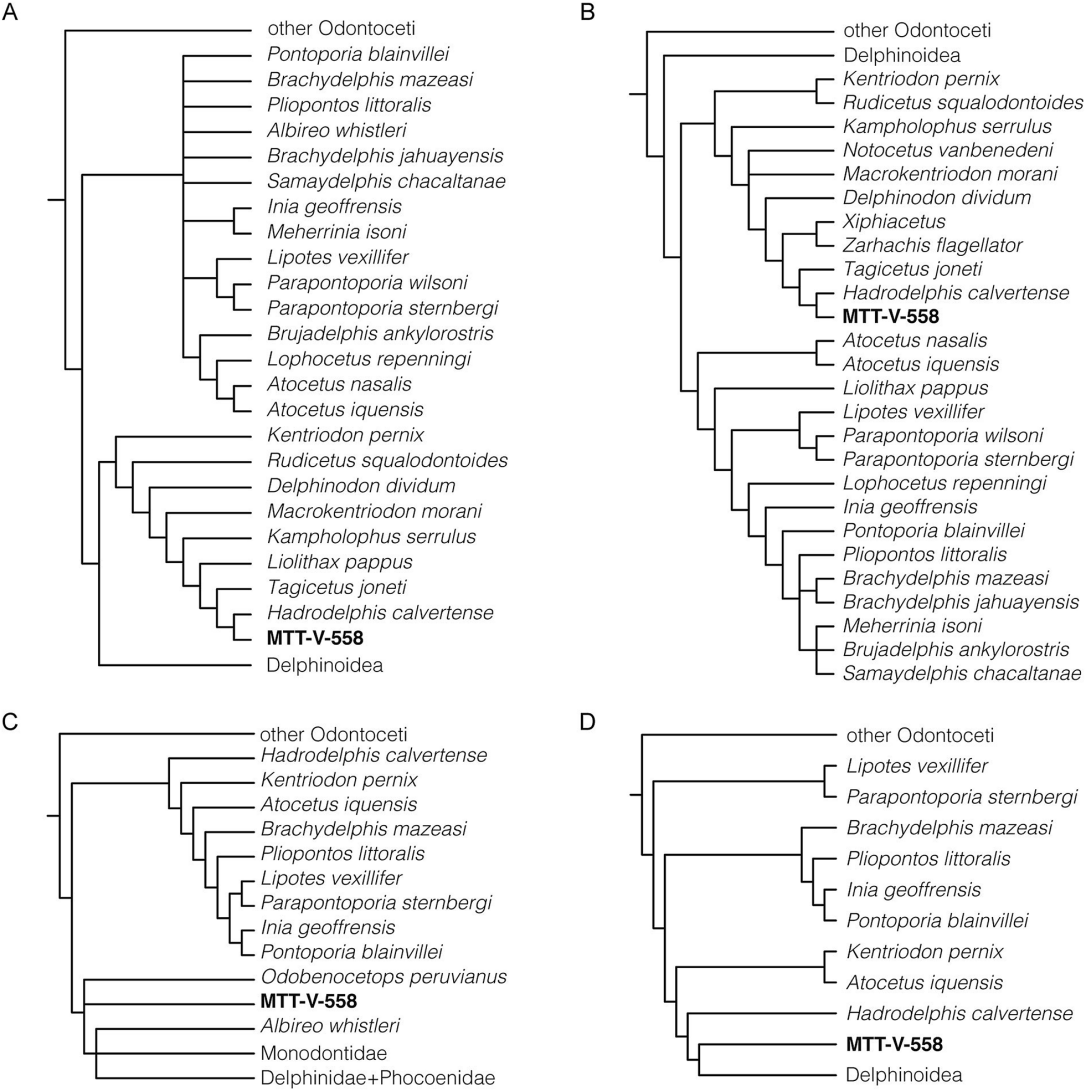
Phylogenetic relationships of the Caujarao delphinidan (MTT-V-558) within Odontoceti. Strict consensus resulting from a single tree with a CI of 0.159 and a RI of 0.565, resulting from a parsimony analysis with a molecular backbone enforced, based on Lambert et al. (2020) (**a**). Strict consensus resulting from 27 trees with a CI of 0.162 and a RI of 0.603, resulting from the analysis omitting non-cranial characters, modified from Lambert et al. (2020) (**b**). Strict consensus resulting from 590 trees with a CI of 0.159 and a RI of 0.565, resulting from a parsimony analysis based on Viglino et al. (2020) (**c**). Strict consensus from 2 trees resulting from the implied weighting (k = 20) after Viglino et al. (2020) with a CI of 0.221 and a RI of 0.629 (**d**)

The first analysis resulted in a single most parsimonious tree, with a CI of 0.159, a RI of 0.565, and a total length of 2038.407 steps (Fig. 5a). The second analysis resulted in 27 most parsimonious trees with a CI of 0.162, a RI of 0.603 and a total length of 1229.56 (Fig. 5b). In both cases, the Caujarao delphinidan MTT-V-558 was recovered as sister taxon to *Hadrodelphis calvertense* and both, to *Tagicetus joneti*; within a clade that includes several other species of early diverging delphinoids.

Within the first analysis, the clade formed by *Tagicetus*, *Hadrodelphis* and MTT-V-558 was recovered on the basis of the following characters: contact of the premaxillae on the anterior third of the rostrum sutured but not fused (c. 9[1]), contact of the premaxillae on the middle third of the rostrum sporadic among the midline (c. 10[1]), lateral margin of the posterior region of rostral edge straight or gently concave in dorsal view (c. 12[0]), lacrimal restricted below the supraorbital process of the frontal (c. 52[0]), lateral edge of premaxilla straight or smoothly curved lacking a inflection of the nasal process (c. 108[0]), occipital shield smoothly convex or concave (c. 156[0]), anterior sinus present but short (c. 158[1]). *Hadrodelphis* was recovered as sister to MTT-V-558 in the basis of: 15 to 17 teeth alveoli completely enclosed in the maxilla (c. 25[4]), infratemporal crest region gently convex (c. 63[1]), rostral basin present (c. 66[1]), premaxillae restricted to the medial position adjacent to the mesorostral canal and nasal opening (c. 75[4]), maxillae laterally to premaxillae exposed anteriorly to the nasal openings (c. 83[0]), nasals in dorsal view with a point on midline and a gap on each side between premaxilla and nasal (c. 117[1]), zygomatic process of squamosal directed anterolaterally (c. 143[1]), in ventral view posterior edge of the paraoccipital process in transverse line with posterior edge of condyle (c. 198[1]).

The second analysis recovered the clade formed by *Tagicetus*, *Hadrodelphis* and MTT-V-558 by: posterior wall of the antorbital notch formed solely by the maxilla (c. 16[0]), dorsal edge of orbit either in line with the edge of the rostrum or slightly above it (c. 48[1]), anterior edge of supraorbital process slightly anterolaterally oriented (c. 50[1]), anterior edge of nasals in line between postorbital process and the anterior tip of the zygomatic process of the squamosal (c. 81[6]), premaxillae overhanging maxillae in the area adjacent to the posterior edge of the nasal openings (c. 111[1]), dorsal surface of nasals medially depressed (c. 119[2]), very shallow squamosal fossa (c. 147[0]), anterior edge of supraoccipital semicircular in dorsal view (c. 153[1]), enlarged preorbital lobe of pterygoid sinus (c. 170[3]), cranial hiatus present (c. 184[1]). MTT-V-558 was recovered as sister taxon to *Hadrodelphis* in the basis of: 15 to 17 alveoli completely enclosed in the maxilla (c. 25[4]), postorbital ridge region gently convex (c. 63[1]), rostral basin present (c. 66[1]), posteriormost end of nasal process of the premaxilla in line with gap between postorbital process and anterior tip of the zygomatic process of the squamosal (c. 75[4]), anterior edge of external bony nares U-shaped (c. 82[1]), zygomatic process of squamosal directed anterolaterally (c. 143[1]), postorbital lobe of pterygoid sinus enlarged and forming a prominent fossa posterior to the optic foramen in the ventral surface of the surface of the supraorbital process of the frontal (c. 171[3]), posterior edge of the paraoccipital process in line with the posterior edge of the condyle in ventral view (c. 198[1]).

The third analysis (Fig. 5c) resulted in 590 most parsimonious trees with a CI of 0.221 and a RI of 0.629, and the fourth (Fig. 5d) in two most parsimonious trees, with a CI of 0.221 and a RI of 0.629. On both analysis MTT-V-558 was recovered as sister taxon to Delphinoidea.

## Discussion and conclusions

The position of MTT-V-558 varied across all four phylogenetic analyses. In the two first analyses (Fig. 5a, b), MTT-V-558 was recovered within a clade sister to Delphinoidea (Fig. 5a) or Inioidea (Fig. 5b). The two subsequent analyses recovered MTT-V-558 as a sister taxon to Delphinoidea (Fig. 5c, d). These positions that have been previously suggested to be occupied by several Miocene delphinoids, where the positions of several taxa have varied among different analyses. In the case of *Tagicetus* and *Hadrodelphis*, their positions have greatly varied, being recovered in different positions across Delphinida. These taxa have been recovered independently either more closely related to Inioidea (Post et al. 2017; Lambert et al. 2020) or to Delphinoidea (Lambert et al. 2018), or stem to both (Lambert et al. 2017b; Post et al. 2017; Peredo et al. 2018). Their affinities have also been disputed, as some analyses recovered them as distantly related (Lambert et al. 2017b, 2020; Post et al. 2017) and others hypothesized them as closely related (Lambert et al. 2018). This variation among phylogenetic analyses can be attributed to the fragmentary state of several specimens, lacking either cranial regions, earbone material or mandibles; but also to the lack of coded postcranial material which could possess some phylogenetic signal (Boessenecker et al. 2020).

The overall cranial morphology of the Caujarao delphinidan displays some characters, commonly seen in the grade ‘Kentriodontidae’ (sensu Barnes 1978). The definition of what a ‘Kentriodontidae’ is and the content of the group have been widely discussed by several authors (Barnes 1985; Muizon 1988b; Peredo et al. 2018; Kimura and Hasegawa 2019), and it is now considered a paraphyletic group composed by several lineages of early diverging delphinidans whose phylogenetic affinities are not yet well elucidated. Despite being recovered in fairly variable positions due to its fragmentary preservation, the Caujarao delphinidan shows some shared features with *Tagicetus* and *Hadrodelphis*. In dorsal view, both *Hadrodelphis* and *Tagicetus* display an open mesorostral groove anterior to the external bony nares as in MTT-V-558. Because the anterior tip of the rostrum in missing in MTT-V-558 it is not possible to assess if the rostrum ended up in a narrow strip as in *Tagicetus* or with a relatively wider profile as in *Hadrodelphis*. The preorbital process of the Caujarao delphinidan is similar to that present in *Atocetus iquensis* and *Atocetus nasalis* due to the shape and orientation of the suture between the frontal, the maxilla and the lacrimal (Muizon 1988b); however, it is not dorso-ventrally expanded on its anterior end as in *Atocetus*. The external bony nares of the Caujarao delphinidan are located at the level of the postorbital process of the frontal; much anteriorly than in other taxa as *Macrokentriodon* or *Hadrodelphis*, where these are located over the level of the zygomatic process of the squamosal (Dawson 1996a, b). The temporal fossa is not as greatly developed as in *Hadrodelphis*, but resembles *Atocetus* both in depth and its overall extent (Muizon 1988b). Unlike other stem delphinidans (e.g., *Rudicetus*; Bianucci 2001), the zygomatic process of the squamosal is not antero-posteriorly elongated, but displays a square-like profile as in more crownward delphinidans. This shape of the zygomatic process should be the result of a fracture; however, it is not clear if the bone was anterio-posteriorly elongated as in most stem delphinidans or if it displayed a shorter profile as in *Tagicetus* (Lambert et al. 2005), due to its abrupt end. Because the dorsalmost part of the vertex is missing (including both nasals) it is not possible to assess the overall contribution from each bone to it. In other taxa like *Delphinus* or *Kentriodon*, the frontal is exposed within the vertex, posterior to the nasal; thus contrasting other stem delphinidans as *Atocetus*, which have a more restricted dorsal exposure of the frontal (Muizon 1988b). Comparisons of other specific cranial characters could lead to ambiguous interpretations, as these could be the result of intraspecific variation. The extension and slight bend of the right premaxilla in the Caujarao delphinidan resembles *Kentriodon nakajimai*, where this extension of the bone has been interpreted to be a result of a secondary deformation. The Caujarao delphinidan could also be secondarily deformed as suggested by the unusual disposition of the premaxilla and the maxilla in the rostrum. However, the lack of a complete vertex of the specimen and other fossil remains in the area impedes the test of this hypothesis. Similar intraspecific variations have been reported in *Atocetus nasalis*, where the number and relative position of the dorsal infraorbital foramina is also diverse within different specimens (Barnes 1985). Because of the preservation state of the specimen (lacking the dorsal end of the vertex, earbones, part of the basicranium and the mandible) it is not possible to assess a more precise identification. Recent phylogenetic analysis (e.g., Peredo et al. 2018; Kimura and Hasegawa 2019; Lambert et al. 2020) evidenced that most of the diagnostic characters that differentiate delphinidan clades or species are found within these regions.

The middle-to-late Miocene fossil record of cetaceans indicates, that along with the global temperature drop by the end of the middle Miocene Climatic Optimum, Odontoceti diversity also entered a phase of change where marine platanistoids (Bianucci et al. 2020) and some lineages of early odontocetes disappeared, being replaced by a more modern community that included the earliest relatives of crown delphinoids, but also a fair diversity of other groups as sperm whales (Lambert et al. 2017a; Benites-Palomino et al. 2020; Collareta et al. 2020), inioids (Lambert et al. 2017b, 2020) and beaked whales (Bianucci et al. 2016). The fossil record indicates that the earliest representatives of crown Delphinida coexisted along with early diverging forms, thus sharing world oceans for at least a couple of millions of years. This has been evidenced on late Miocene fossils from both Japan (Kimura and Hasegawa 2019, 2020) and Peru (Muizon 1988b; Lambert and Muizon 2013), where early diverging delphinidans have been found along with more crownward delphinidans. Nevertheless, the factors that drove the final turnover event in which modern Delphinida prevailed and stem delphinidans disappeared, is still unknown.

The fossil record of cetaceans from the Caribbean has been improved during the last 10 years, specially due to the description of the kogiid *Nanokogia isthmia* and the inioid *Isthminia panamensis*, from the late Miocene Chagres Fm. in Panama (Pyenson et al. 2015; Velez-Juarbe et al. 2015). The fossil record from Southern Caribbean in the vicinity of the gulf of Venezuela in both Colombia and Venezuela, indicates that there was a fair diversity of taxa, including inioids, squalodelphinids and baleen whales inhabiting proto-Caribbean waters during the Miocene (Aguirre-Fernández et al. 2017a, b). Despite its fragmentary state, the Caujarao delphinidan is among the best-preserved specimens of the region so far and indicates that stem delphinidans were also present in the region during the early late Miocene. The preservation state of the specimen indicates that the region in northern Venezuela could yield new findings in the near future, thus helping to understand the evolutionary history of cetaceans in southern Caribbean. Nevertheless, future prospection work in the area will be needed in order to fully support this hypothesis.

## Data Availability

The fossil specimen MTT-V-558 is available at the Museo Ángel Segundo López, Taratara, Falcón State. All data generated or analyzed during this study are included in this published article.
